# Silver-Russell Syndrome: A Case Report

**DOI:** 10.1186/1757-1626-1-304

**Published:** 2008-11-09

**Authors:** Sunil Kumar, AP Jain, Sachin Agrawal, Sindu Chandran

**Affiliations:** 1 Department of Medicine, Mahatma Gandhi Institute of Medical Sciences, Sewagram, Wardha, (Maharashtra), India

## Abstract

A 15-year-old male boy with hemihypertrophy (left side) of the body was admitted in the hospital with the history of repeated attacks of convulsion. The patient was diagnosed as Silver-Russell syndrome on clinical ground. Silver-Russell syndrome (SRS) is a very rare genetic disorder that appears no later than early childhood. This is usually characterized by asymmetry in the size of the two halves or other parts of the body. Silver-Russell Syndrome occurs mostly in isolated cases because of sporadic genetic changes (mutations) for no apparent reason. For lack of facilities we were not able to do genetic study.

## Case Presentation

A 15-year-old boy Hindu tribal from a rural area of Wardha district, Maharastra, India, a product of nonconsanguineous marriage, with a history of congenital short stature presented in emergency unit of medicine department with status epilepticus. He had one more brother with normal body habitus enjoying normal life. Boy's parent gave history of seizure disorder since one month without any anti epileptic drugs. His seizure was managed by giving intravenous phenytoin sodium loading dose, then maintained on phenytoin 100 mg thrice daily. He had low birth weight, feeding difficulties, short stature, and limb asymmetry. He has had a poor appetite since childhood. Despite difficulties, he is attending his school regularly.

**Figure 1:**
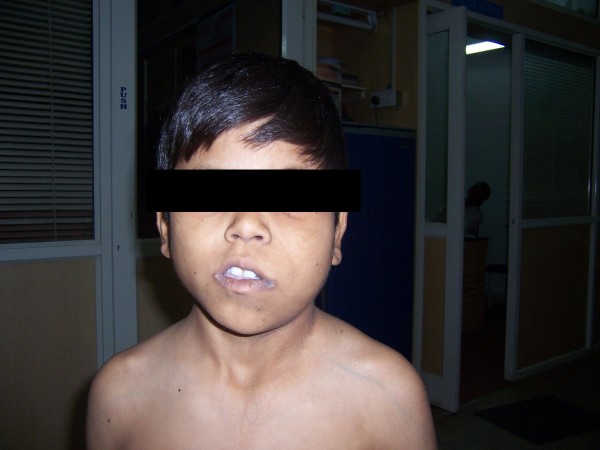
Shows the phenotypic appearance of the baby – the hemihypertrophy of the left side of the body including face.

**Figure 2:**
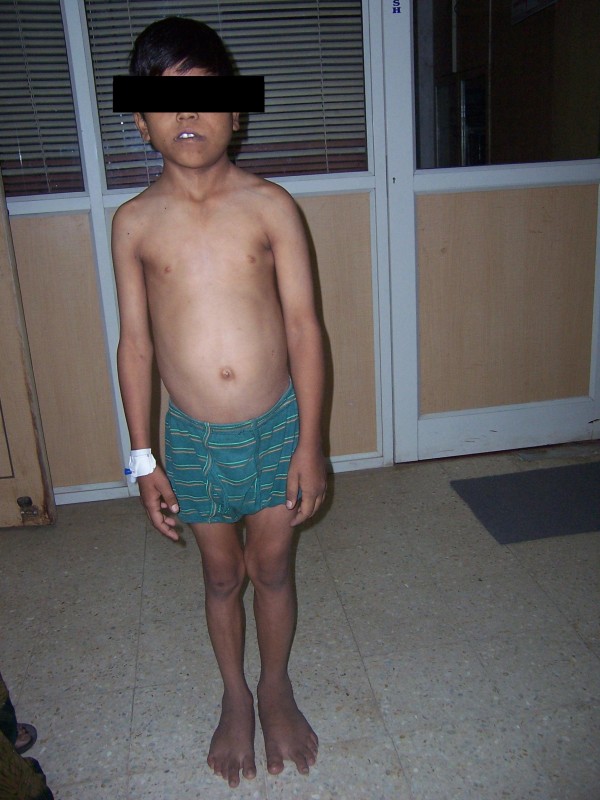
Shows the phenotypic appearance of the baby – the hemihypertrophy of the left side of the body including face.

**Figure 3:**
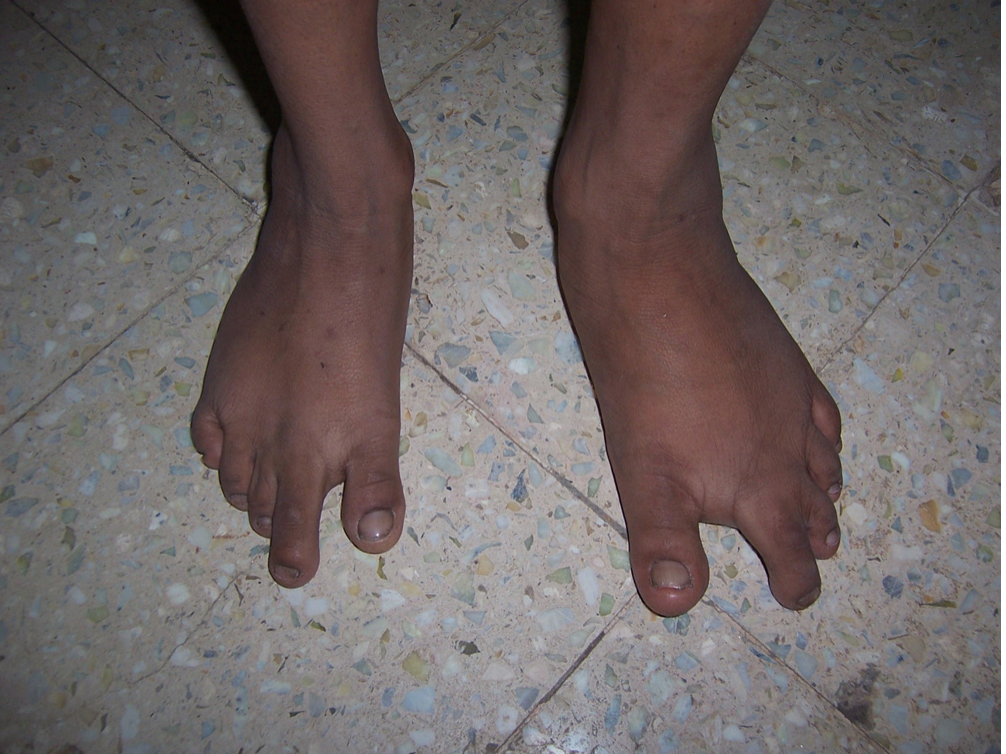
Clinodactyly of the 2^nd ^and 3^rd ^toe.

On physical examination, he was well-appearing, thin, and short with normal head circumference, with a broad forehead, triangular facies, low-set prominent ears, and crowded teeth. The third and fourth phalanges of the feet showed clinodactyly. Asymmetry of the hands, phalanges, and lower extremities were noted, with hemihypertrophy of the left lower extremity (fig 1, 2, 3). His height was 97 cm which was low as per his age and arm span was 83 cm. No cafe-au-lait macules were noted.

All basic investigation was within normal unit. CT scan brain was normal but his EEG showed abnormal spikes in right frontoparietal area. Chromosome analysis could not be done because of infeasibility and lack of facility. Intelligence and psychomotor development were normal as per his socioeconomic status.

## Discussion

Russell-Silver syndrome is a disorder present at birth that involves poor growth, low birth weight, short height, and differences in the size of the two sides of the body. In 1953, Silver et al reported two unrelated children with congenital hemihypertrophy, low birth weight, and short stature. In 1954, Russell described five unrelated children with extreme intrauterine growth retardation and characteristic facial features. These children had remained small and in two cases there was body asymmetry. The characteristic features described were triangular shaped face with a broad forehead and pointed, small chin, together with a wide, thin, 'shark-like' mouth. Although each author emphasised rather different features, the composite features have been identified as the Russell-Silver syndrome, and attempts to separate the Silver syndrome from the Russell syndrome, depending on whether asymmetry is present or absent, have not generally been accepted [1].

It is estimated that 7–10% of patients with this syndrome have a defect in a gene called the maternal uniparental disomy (UPD) for chromosome 7. However, a cause cannot be identified for most patients. Most cases occur in a person whose family has no history of the disease. Incidence ranges from 1 in 3,000 to 1 in 100,000 and worldwide more than 500 cases have been reported with equal male-to female ratio[2]. Clinically apparent limb asymmetry occurs in 60% of patients reported. Although Silver et al originally described this feature as hemihypertrophy, it has been unclear whether the asymmetry is the result of hemihypertrophy, hemiatrophy, or a disturbance of the normal range of symmetry[3]. There have been few measurements of limb length to resolve this issue. Tanner et al[4] in a careful anthropometric study of limb length in 39 subjects with Russell-Silver syndrome found a continuity in the variation of limb length between the normal controls and those with the Russell-Silver syndrome, leading them to the conclusion that the cause of the asymmetry is a disturbance in the control of symmetry.

Normal intelligence is the rule in this syndrome. There may, however, be some delay in the early motor milestones owing to the decreased muscle bulk and relatively large head.

## Consent

Written informed consent was obtained from the patient for publication of this case report and accompanying images. A copy of the written consent is available for review by the Editor – in – Chief of this Journal.

## Competing interests

The authors declare that they have no competing interests.

## Authors' contributions

SK was responsible for patient care, follow up, preparing manuscript and drafting of paper. APJ was head of department and responsible for patient care. SA and SC collected the data and involved in the radiological investigation of the study. All authors read and approved the final manuscript.
